# Supplementary material for: Reduced Levels of ABCA1 Transporter Are Responsible for the Cholesterol Efflux Impairment in β-Amyloid-Induced Reactive Astrocytes: Potential Rescue from Biomimetic HDLs
Source: Int J Mol Sci. 2021 Dec 22;23(1):102. doi: 10.3390/ijms23010102 (PMC8745016; doi:10.3390/ijms23010102)
Supplement: Supplementary file 1 [file ijms-23-00102-s001.zip › ijms-1448826-supplementary.pdf]

Supplementary Materials

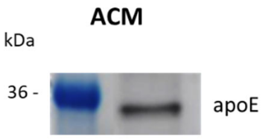

Figure S1. Detection of apoE in ACM by SDS-PAGE/WB. ACM was obtained by collecting the astrocyte culture medium after 48 hours of growth. After filtration, the presence of apolipoprotein E (apoE) was evaluated by SDS-PAGE/WB.

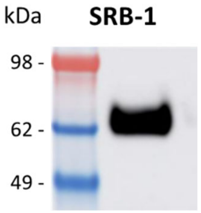

Figure S2. Detection of SRB-1 in hCMEC/D3 by SDS-PAGE/WB. Cells were lysed and the presence of SR-B1 receptor was evaluated by SDS-PAGE/WB.
